# Metabotropic Glutamate Receptor 5 and 8 Modulate the Ameliorative Effect of Ultramicronized Palmitoylethanolamide on Cognitive Decline Associated with Neuropathic Pain

**DOI:** 10.3390/ijms20071757

**Published:** 2019-04-09

**Authors:** Serena Boccella, Ida Marabese, Monica Iannotta, Carmela Belardo, Volker Neugebauer, Mariacristina Mazzitelli, Gorizio Pieretti, Sabatino Maione, Enza Palazzo

**Affiliations:** 1 Department of Experimental Medicine, Pharmacology Division, University of Campania “L. Vanvitelli”, 80138 Naples, Italy; boccellaserena@gmail.com (S.B.); ida.marabese@unicampania.it (I.M.); monica.iannotta@gmail.com (M.I.); carmelabelardo@libero.it (C.B.); sabatino.maione@unicampania.it (S.M.); 2 Department of Pharmacology and Neuroscience, School of Medicine, Texas Tech University Health Sciences Center, Lubbock, TX 79430-6592, USA; volker.neugebauer@ttuhsc.edu (V.N.); mariacristina.mazzitelli@ttuhsc.edu (M.M.); 3 Garrison Institute on Aging, Texas Tech University Health Sciences Center, Lubbock, TX 79430-6592, USA; 4 Center of Excellence for Translational Neuroscience and Therapeutics, Texas Tech University Health Sciences Center, Lubbock, TX 79430-6592, USA; 5 Department of Plastic Surgery, University of Campania “L. Vanvitelli”, 80138 Naples, Italy; gorizio.pieretti@unicampania.it

**Keywords:** spared nerve injury, discriminative memory, long term potentiation, lateral entorhinal cortex-dentate gyrus, ultramicronizedpamitoylethanolamide, metabotropic glutamate receptor 5 and 8

## Abstract

This study investigated whether metabotropic glutamate receptor (mGluR) 5 and 8 are involved in the effect of ultramicronizedpalmitoylethanolamide (um-PEA) on the cognitive behavior and long term potentiation (LTP) at entorhinal cortex (LEC)-dentate gyrus (DG) pathway in mice rendered neuropathic by the spare nerve injury (SNI). SNI reduced discriminative memory and LTP. Um-PEA treatment started after the development of neuropathic pain had no effects in sham mice, whereas it restored cognitive behavior and LTP in SNI mice. 2-Methyl-6-(phenylethynyl) pyridine (MPEP), a selective mGluR5 antagonist, improved cognition in SNI mice and produced a chemical long term depression of the field excitatory postsynaptic potentials (fEPSPs) in sham and SNI mice. After theta burst stimulation (TBS) MPEP restored LTP in SNI mice. In combination with PEA, MPEP antagonized the PEA effect on discriminative memory and decreased LTP in SNI mice. The (RS)-4-(1-amino-1-carboxyethyl)phthalic acid (MDCPG), a selective mGluR8 antagonist, did not affect discriminative memory, but it induced a chemical LTP and prevented the enhancement of fEPSPs after TBS in SNI mice which were treated or not treated with PEA. The effect of PEA on LTP and cognitive behavior was modulated by mGluR5 and mGluR8. In particular in the SNI conditions, the mGluR5 blockade facilitated memory and LTP, but prevented the beneficial effects of PEA on discriminative memory while the mGluR8 blockade, which was ineffective in itself, prevented the favorable action of the PEA on LTP. Thus, although their opposite roles (excitatory/inhibitory of the two receptor subtypes on the glutamatergic system), they appeared to be required for the neuroprotective effect of PEA in conditions of neuropathic pain.

## 1. Introduction

Sensory and cognitive disorders are comorbidities of neuropathic pain in both, humans and rodents [1]. The synaptic changes associated with chronic pain states have mainly been focused at the peripheral and spinal dorsal horn level [2,3]. Supraspinal areas such as the hippocampus have remained less explored, despite its ascertained role as anatomical substrate of the interactions between chronic pain and cognitive or retention memory disorders [4,5,6,7,8]. Indeed, chronic pain deeply affects the neural activity [9,10], mammalian target of rapamycin (mTOR) signaling [11], synaptic plasticity at dentate gyrus (DG), CA1 or CA3 synapses [6,12] and neurogenesis in the DG [13]. Hippocampus receives strong cortical inputs from the entorhinal cortex via the perforant pathway [14]. The contribution of this flow of information is critical for integrating emotionally salient information associated with spatial-temporal orientation and place memorization [15]. As a result of this, cognitive decline associated with neuropathic pain could be associated with entorhinal cortex-hippocampal signaling perturbation and neuroplasticity deficit. Among the pharmacological strategies for sensory and affective/cognitive disorders associated with neuropathic pain, palmitoylethanolamide (PEA), an endocannabinoid anandamide congener, has shown antinflammatory, analgesic, immunomodulatory and neuroprotective effects and to reverse cognitive impairments associated with several chronic pain conditions [16,17,18] by restoring glutamatergic transmission homeostasis [16,17,19,20,21,22]. Elevated glutamate signaling is implicated in the etiology and progression of neuropathic pain and its neuropsychiatric comorbidities [21,22,23]. Consequently, approaches aimed at correcting glutamate hyperactivity could be beneficial as therapy for neuropathic pain and its psychiatric consequences, or they could potentiate the effect of analgesic drugs. Allosteric modulators of metabotropic glutamate receptor subtype 5 and 8 (mGluR5 and mGluR8) have shown analgesic and antidepressant effects [24,25,26,27,28,29,30]. Moreover, it has also been shown that mGluR activation appears indispensable for the endocannabinoid analgesic/neuroprotective action [31,32,33,34,35] and the endocannabinoid-like PEA restored neuropsychiatric behavior by modulating excitatory synapse homeostasis in the medial prefrontal cortex [17]. On this basis, we undertook the current study with the aim of assessing the effect of mGluR5 and mGluR8 blockade on cognitive behavior and long term potentiation (LTP) at the lateral entorhinal cortex (LEC)-dentate gyrus (DG) pathway, and whether the beneficial effects of ultramicronized (um)- PEA are affected by mGluR5 and mGluR8 blockade in neuropathic pain conditions. Shaping glutamate transmission malfunctioning through mGluR manipulation may represent a strategy for ameliorating the beneficial effects of neuroprotective agents such as PEA in chronic and untreatable pain conditions. 

## 2. Results

### 2.1. Discriminative Memory

**Figure 1:**
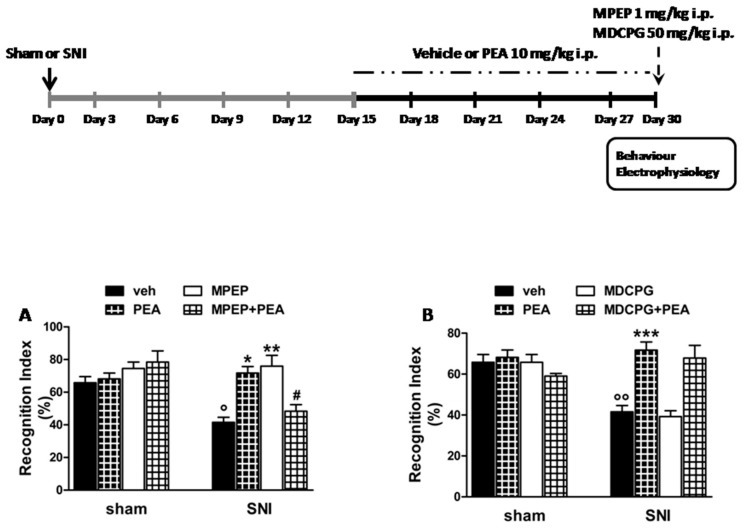
Effect of a single administration of vehicle, MPEP (1 mg/kg, i.p.), MDCPG (50 mg/kg, i.p.) on discriminative memory in sham and SNI mice receiving a 15-day treatment with vehicle or PEA (10 mg/kg, i.p.) 15 day after the sham or SNI surgery (day 0). The upper panel shows the experimental design and timeline of the sham or SNI surgery, the chronic treatment with vehicle or PEA and the single administration of vehicle, MPEP or MDCPG. (**A**,**B**) show the effect of vehicle, MPEP (**A**) or MDCPG (**B**) on the recognition index (RI) in sham or SNI receiving a chronic treatment with vehicle or PEA (10 mg/kg, i.p.). The administration of vehicle MPEP or MDCPG was carried out 1 h before the acquisition trial. Data are represented as the mean ± SEM of six mice per group. ° indicates significant differences vs.sham/veh, * indicates significant differences vs. SNI/veh and **^#^** indicates significant differences vs.SNI/PEA in SNI mice. *p* < 0.05 was considered statistically significant.

In the novel object recognition test, (spared nerve injury) SNI mice that received a chronic treatment with vehicle (Figure 1A) showed a significant decrease in the recognition index (RI) (41.6 ± 3.1%, one way-ANOVA followed by Bonferroni post-hoc test) compared with sham mice that received chronic treatment with vehicle (65.8 ± 3.8%) (Figure 1B). A 15 day treatment with PEA (10 mg/kg, i.p.) did not change the RI in the sham mice (68.2 ± 3.6%) whereas it significantly increased it in SNI mice (71.7 ± 4.0%, F_(7,38)_= 7.33; *p* < 0.0001). A single administration of 2-methyl-6-(phenylethynyl)pyridine,(MPEP, 1 mg/kg, i.p.), a selective mGluR5 antagonist, was devoid of effect in sham mice (74.5 ± 3.9%) whereas it increased the RI in SNI mice (75.9 ± 6.6%) (Figure 1A). MPEP also antagonized the effect of PEA in SNI mice (48.4 ± 4%) although not in the sham mice (78.4 ± 6.8%). A single administration of (RS)-4-(1-amino-1-carboxyethyl)phthalic acid (MDCPG, 50 mg/kg, i.p.), a selective mGluR8 antagonist, did not change the RI in sham (65.7 ± 3.8%) and SNI mice (39.3 ± 2.8%), treated or not with PEA (59.1 ± 1.23% and 67.8 ± 6.2%, respectively) (Figure 1B).

### 2.2. Effect of MPEP on LTP at the LEC-DG Pathway in Sham Mice Treated with Vehicle or PEA

**Figure 2:**
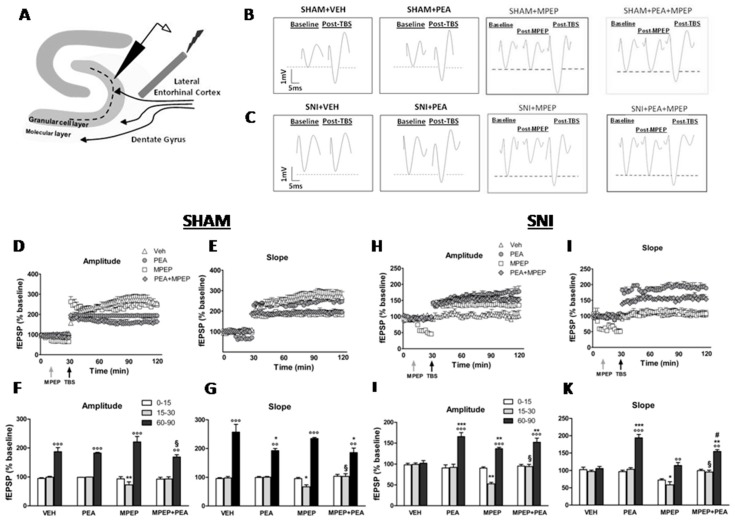
Effect of intra-LEC microinjection of vehicle or MPEP (5 nmol) on the LTP at the LEC-DG pathway in sham and SNI mice treated with vehicle or with PEA (10 mg/kg, i.p.) for two weeks. (**A**) shows a schematic illustration of electrode placements with the stimulating electrode in the LEC for stimulating the lateral perforant path (LPP) fibers and microinjecting drug or vehicle solutions and the recording electrode in the DG for recording the fEPSPs of the granular cells. (**B**,**C**) show sample traces of a single evoked fEPSP recorded in the DG during baseline, after MPEP microinjection into the LEC or TBS application in both, sham (**B**) or SNI (**C**) mice treated with vehicle or PEA. (**D**–**F**) show time-dependent changes in the amplitude (**D**,**H**) and slope (**E**,**I**) of fEPSPs after vehicle or MPEP (5 nmol) microinjection in vehicle or PEA-treated sham (**D**,**E**) or SNI (**H**,**I**) mice. (**F**,**G**,**J**,**K**) show the average of normalized amplitude (**F**,**J**) and slope (**G**,**K**) of fEPSPs at different time points (0–15, 15–30 and 60–90 min) after microinjection of MPEP (15–30) or TBS application (60–90) normalized to basal responses in vehicle or PEA-treated sham (**F**,**G**) or SNI (**J**,**K**) mice. Data are represented as mean ± SEM of the amplitude and slope of fEPSPs. **^°^** Indicate significant differences vs. pre-TBS (15-30 min), * indicates significant differences vs. vehicle-treated mice, ^#^ indicates significant differences vs. PEA-treated mice and ^§^ indicate significant differences vs. MPEP microinjection. *p* < 0.05 was considered statistically significant. Vertical and horizontal scale bars indicate 1mV and 5ms, respectively. Grey arrow indicates intra-LEC MPEP microinjection and black arrow the TBS application.

The theta-burst stimulation (TBS) was applied in the LEC 30 min after the registration of the baseline of field excitatory postsynaptic potentials (fEPSPs) in the DG (Figure 2A). In sham mice treated with vehicle, the TBS induced a LTP (Figure 2B) associated with a significant increase in both the amplitude (60–90 min: 186.87 ± 14.32% vs. 15–30 min: 99.49 ± 2.93%) (degrees of freedom, F_2,42_ = 221; *p* < 0.0001) and slope (60–90 min: 257.27 ± 27.15% vs. 15–30 min: 97.87 ± 5.0%) (F_2,42_ = 221; *p* < 0.0001) (F_2,42_ = 199; *p* < 0.0001) of the fEPSPs as revealed by one-way ANOVA followed by Dunnet’s post hoc test (Figure 2D–G). The chronic treatment with PEA (10 mg/kg, i.p.) did not modify the amplitude (60–90 min: 182.30 ± 2.47%) (F_2,42_ = 187.2; *p* < 0.0001) and the slope (60–90 min: 192.44 ± 7.55%) (F_2,42_ = 222.4; *p* < 0.01) of the fEPSP in vehicle-treated sham mice as indicated by two-way ANOVA followed by the Bonferroni post-hoc test (Figure 2D–G). The microinjection of MPEP (5 nmol) into the LEC did not affect the magnitude of LTP induced by TBS stimulation. Indeed, the amplitude (60–90 min: 220.87 ± 18.63%) (t_5_ = 3.24; *p* < 0.0001) and the slope (60–90 min: 234.49 ± 3.74%) (t_5_ = 3.13; *p* < 0.0001) of the fEPSPs remained unchanged compared with vehicle-treated sham mice (Figure 2D–G). However, MPEP reduced both, the amplitude (15–30 min: 72.97 ± 10.15% vs. 0–15min: 93.67 ± 7.54%) (t_5_ = 4.2; *p* < 0.01) and slope values (15–30 min: 66.95 ± 7.0% vs. 0–15 min: 95.66 ± 4.03%) (t_5_ = 2.7; *p* < 0.05) of basal fEPSPs in sham mice. When MPEP (5 nmol) was administered in sham mice chronically treated with PEA, the amplitude (60–90 min: 169.3 ± 7.28% vs. 15–30 min: 93.34 ± 7.38%) (t_5_ = 7.88; *p* < 0.0001) and slope (60–90 min: 185.74 ± 15.53% vs. 15–30 min: 103.31 ± 9.27) (t_5_ = 5.58; *p* < 0.0001) of the fEPSPs post-TBS remained unchanged compared to vehicle-treated sham mice (Figure 2D–G). However, the observed decrease in both the amplitude and slope of the basal fEPSPs was no more present in sham mice chronically treated with PEA which received a single MPEP microinjection (Figure 2D–G).

### 2.3. Effect of MPEP on LTP at the LEC-DG Pathway in SNI Mice Treated with Vehicle or with PEA

Vehicle-treated SNI mice did not show LTP of the fEPSPs after TBS (Figure 2C). Indeed, both the amplitude (60–90 min: 102.21 ± 6.17% vs. 15–30 min: 99.2 ± 3.71%) (F_2,42_ = 43.2; *p* > 0.05) and slope (60–90 min: 105.7 ± 5.99% vs. 15–30 min: 97.1 ± 4.1%) (F_2,42_ = 47.6; p> 0.05) of the fEPSPs did not change after TBS, as assessed by one-way ANOVA followed by Dunnet’s post hoc test (Figure 2H–K). The chronic treatment with PEA significantly rescued the LTP in SNI mice. Indeed, two-way ANOVA for repeated measures followed by the Bonferroni post-hoc test revealed significant differences in the amplitude (60–90 min: 165.64 ± 9.54% vs. 15–30 min: 92 ± 7.73%) (F_2,42_ = 194; *p* < 0.0001) and slope (60–90 min: 193.87 ± 9.85% vs. 15–30 min: 103.64 ± 4.65%) (F_2,42_ = 218.6; *p* < 0.01) of EPSPs after TBS as compared to vehicle-treated SNI mice (Figure 2H–K). As observed in sham mice, the microinjection of MPEP (5 nmol) reduced the amplitude and slope of basal fEPSPs also in SNI mice. Indeed, the amplitude (15–30 min: 52.61 ± 3.69% vs. 0–15 min: 90.34 ± 3.13%) (t_5_ = 4.18; *p* < 0.01) and the slope (15–30 min: 58.68 ± 8.92% vs. 0–15 min: 72.37 ± 3.68%) (t_5_ = 3.98; *p* < 0.01) of the fEPSPs before the application of the TBS significantly decreased as assessed by unpaired t-student test (Figure 2H–K). By doing so, MPEP rescued the LTP. Indeed, it significantly increased both the amplitude (60–90 min: 136.58 ± 3.17%) (t_5_ = 3.18; *p* < 0.01) and slope (60–90 min: 114.46 ± 8.57%) (t_5_ = 3.47; *p* < 0.01) of fEPSPs after TBS in SNI mice. When MPEP (5 nmol) was microinjected into the LEC of SNI mice treated with PEA the amplitude (15–30 min: 94.3 ± 5.5.0% vs. 0–15 min: 95.4 ± 3.78%) (t_5_ = 1.84; *p* = 0.55) and the slope (15–30 min: 95.77 ± 5.0% vs. 0–15 min: 99.76 ± 3.78%) (t_5_ = 1.55; *p* = 0.34) of basal fEPSPs did not change (Figure 2H–K) as well as it did not change both, the amplitude (60–90 min: 152.1 ± 9.82%) (t_5_ = 4.23; *p* < 0.01) and slope (60–90 min: 154.39 ± 5.21%) (t_5_ = 4.12; *p* < 0.01) of fEPSPs after TBS (Figure 2H–K).

### 2.4. Effect of MDCPG on LTP at the LEC-DG Pathway in Sham Mice Treated with Vehicle or with PEA

**Figure 3:**
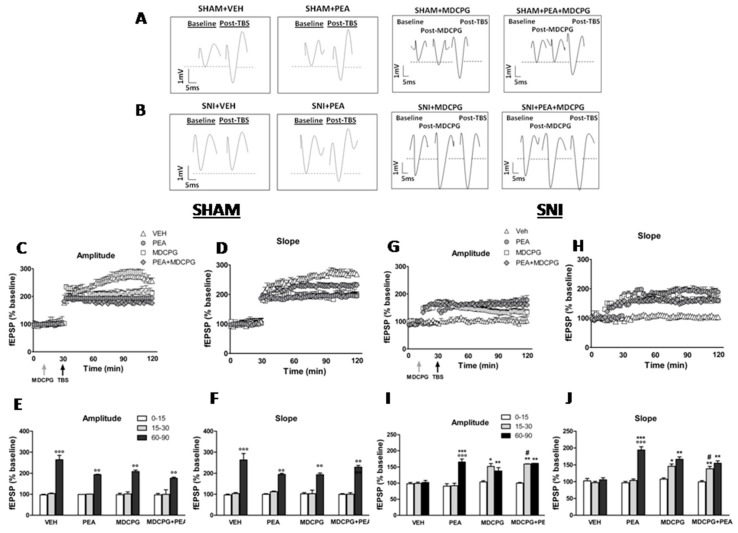
Effect of intra-LEC microinjection of vehicle or MDCPG (100 nmol) on the LTP at the LEC-DG pathway in sham and SNI mice treated with vehicle or with PEA (10 mg/kg, i.p.) for two weeks. (**A**,**B**) show sample traces of a single evoked fEPSP recorded in the DG during baseline, after MDCPG (100 nmol) microinjection into the LEC or TBS application in both, sham (**A**) or SNI (**B**) mice treated with vehicle or PEA. (**C**,**D**,**G**,**H**) show time-dependent changes in the amplitude (**C**,**G**) and slope (**D**,**H**) of fEPSPs after vehicle or MDCPG (100 nmol) microinjection in vehicle or PEA-treated sham (**C**,**D**) or SNI (**G**,**H**) mice. (**E**,**F**,**I**,**J**) show the average of normalized amplitude (**E**,**I**) and slope (**F**,**J**) of fEPSPs at different time points (0–15, 15–30 and 60–90 min) after microinjection of MDCPG (15–30) or TBS application (60–90) normalized to basal responses in vehicle or PEA-treated sham (**E**,**F**) or SNI (**I**,**J**) mice. Data are represented as mean ± SEM of the amplitude and slope of fEPSPs. **^°^** Indicate significant differences vs. pre-TBS (15–30 min), * indicates significant differences vs. vehicle-treated mice, ^#^ indicates significant differences vs. PEA-treated mice and ^§^ indicate significant differences vs.MDCPG microinjection. *p* < 0.05 was considered statistically significant. Vertical and horizontal scale bars indicate 1mV and 5ms, respectively. Grey arrow indicates intra-LEC MDCPG microinjection and black arrow indicates TBS application.

As already mentioned, the application of TBS into the LEC produced a LTP of the fEPSPs in the DG in sham mice treated with vehicle or with PEA (Figure 3A). The microinjection of MDCPG (100 nmol) into the LEC did not affect the LTP of fEPSPs in the DG of sham mice. Indeed, both the amplitude (60–90 min: 208.61 ± 7.0% vs. 15–30 min: 101.91 ± 9.49%) (t_5_ = 4.7; *p* < 0.0001) and the slope (60–90 min: 193.61 ± 8% vs. 15–30 min: 102.73 ± 16.65%) (t_5_ = 5.28; *p* < 0.0001) of the fEPSPs remained unchanged in vehicle-treated sham mice, as revealed by unpaired t-student test (Figure 3C–F). The microinjection of MDCPG (100 nmol) did also not affect the LTP of both, the amplitude (60–90 min: 176.9 ± 5.0% vs. 15–30 min: 99.73 ± 21.85%) (t_5_ = 6.62; *p* < 0.0001) and the slope (60–90 min: 229.53 ± 8.26% vs. 15–30 min: 99.99 ± 6.99%) (t_5_ = 7.5; *p* < 0.0001) of the fEPSPs in PEA-treated sham mice as revealed by unpaired t-student test (Figure 3C–F).

### 2.5. Effect of MDCPG on LTP at the LEC-DG Pathway in SNI Mice Treated with Vehicle or PEA

As already mentioned, the application of the TBS into the LEC did not produce LTP of fEPSPs in the DG of vehicle-treated SNI mice (Figure 3B). The chronic treatment with PEA rescued the LTP of fEPSPs in SNI mice (Figure 3G–J). Intriguingly, the microinjection of MDCPG (100 nmol) into the LEC significantly potentiated the basal fEPSPs in the DG of vehicle-treated SNI mice. Indeed, both the amplitude (15–30 min: 152.07 ± 8.92% vs. 0–15 min: 103.67 ± 3.68%) (t_5_ = 7.14; *p* < 0.01) and the slope (15–30 min: 145.86 ± 6.85% vs. 0–15 min: 107.63% ± 3.76%) (t_5_ = 6.54; *p* < 0.05) of the basal fEPSPs significantly increased as assessed by unpaired t-student test (Figure 3G–J). Similarly, the microinjection of MDCPG (100 nmol) increased both, the amplitude (15–30 min: 159.56 ± 0.46% vs. 0–15 min: 99.9 ± 2.27%,) (t_5_ = 7.32; *p* < 0.05) and the slope (15–30 min: 138.7 ± 6.85% vs. 0–15 min: 99.49 ± 3.76%,) (t_5_ = 7.84; *p* < 0.05) of the basal fEPSPs in SNI mice chronically treated with PEA (Figure 3G–J). By doing so, the application of the TBS did not further potentiate the amplitude (60–90 min: 137.78 ± 10.7%) (t_5_ = 8.22; *p* < 0.0001) and the slope (60–90 min: 166.6 ± 7.27%) (t_5_ = 9.0; *p* < 0.0001) of basal fEPSPs in vehicle-treated SNI mice which received a single microinjection of MDCPG (100 nmol). Similarly, the application of the TBS did not further potentiated the amplitude (60–90 min: 161.49 ± 0.35%) (t_5_ = 8.22; *p* < 0.0001) and the slope (60–90 min: 155 ± 7.27%) (t_5_ = 9.0; *p* < 0.0001) of fEPSPs in PEA-treated SNI mice (Figure 3G–J).

## 3. Discussion

### 3.1. Discriminative Memory and LTP at the LEC-DG Pathway in SNI Mice

Affective and cognitive impairments are comorbidities often found in neuropathic pain states in both, humans and rodents [1,36]. Consistently, in the current study, mice showed a deficit in discriminative memory after the SNI of the sciatic nerve. The entorhinal cortex-DG is the main pathway by which cortical sensory information reach the hippocampus and constitutes the neural substrate involved in the processing of episodic memory. In particular, the LEC-DG pathway processes the non-spatial episodic memory [37]. The loss of functional connectivity within this circuitry could be the basis of the impairments in discriminative memory, such as those related to chronic pain [6,38,39]. Consistently, we found in the current study, as observed in a previous one [16], that the SNI increased the slope and amplitude of basal single pulse of the fEPSPs and disrupted the LTP after TBS application. Plausibly, this increase in the synaptic responses before TBS would prevent the further enhancement of the amplitude and slope of fEPSPs occluding the LTP in a sort of saturation [40,41]. The loss of LTP associated with memory deficits after SNI in rodents has already been found in the hippocampus at the CA3–CA1 and LEC-DG synapses [7,16,41]. These deficits also proved to be associated with glutamate elevation [16,41], whereas the levels of GABA remained unmodified in the DG of SNI mice, possibly as a homeostatic mechanism to counteract the altered glutamatergic tone [16,17]. Inappropriate brain excitability within certain brain circuitries is proposed as a key mechanism contributing to neuropathic pain [19,42,43] and other neuropsychiatric disorders [44,45]. The elevation in glutamate could also cause neuroplasticity affecting group III mGluRs [46] as protective mechanism to counteract abnormal glutamate in pathological conditions. An increase in glutamate level and mGluR8 expression has indeed been found in the central nucleus of the amygdala, and in the dorsal striatum in chronic pain conditions [47,48].

### 3.2. The Effect of a Chronic Treatment with PEA on Discriminative Memory and LTP at the LEC-DG Pathway in Sham and SNI Mice

The chronic treatment with PEA rescues the discriminative memory and LTP at the LEC-DG pathway in SNI mice. Accordingly, the chronic treatment with PEA has already shown to revert mechanical allodynia, [16], thermal hyperalgesia, discriminative and spatial memory [16,17], glutamate levels in the DG and medial prefrontal cortex [16,17] and glutamatergic synapse homeostasis in the medial prefrontal cortex [17] in SNI mice.

Due to the role of glutamate in memory formation in the hippocampus, the normalization of glutamatergic transmission could be at the base of the PEA recovery on LTP and cognitive behavior [49].

### 3.3. The Role of mGluR5 on the Effect of PEA on Discriminative Memory and LTP at the LEC-DG Pathway in Sham and SNI Mice

The contribution of mGluR5 on LTP in neuropathic pain conditions associated with cognitive impairments has never been investigated and remains controversial [50,51,52,53,54]. In the current study, a single administration of MPEP 30 days after the consolidation of neuropathic pain, improved the discriminative memory and restored the disruption of the LTP. In particular, MPEP induced a chemical long term depression decreasing the slope and amplitude of the basal fEPSPs which resulted in an increase, following SNI. This would suggest a tonic role of the mGluR5 in guaranteeing the basal maladaptative excitatory transmission in this pathological condition. In normal conditions, the activation of mGluR5 is required for LTP, also due to its contribution in rising the intracellular Ca^2+^ [55]. In neuropathic pain conditions, a dysregulation of Ca^2+^ is reported, and it contributes to pain-related cognitive disorders [56,57]. Pertinent to this, the blockade of mGluR5 would be favorable. Indeed, the MPEP-induced chemical long term depression permitted the recovery of LTP after TBS in SNI mice. It has been reported that MPEP restored LTP in the DG in pathological conditions through an attenuation of the effect of interleukin-18 (IL-18), a pro-inflammatory cytokine whose level is elevated in pathological states associated with learning and memory impairments [52]. Consistently, IL-18 stimulates glutamate release and enhances postsynaptic responses in CA1 pyramidal neurons, thereby facilitating basal synaptic transmission [41]. Intriguingly, when MPEP was administered in SNI mice chronically treated with PEA, the effects of MPEP in facilitating discriminative memory and decreasing the amplitude and slope of basal fEPSPs were nullified. At first sight, these results seem to contradict each other because both MPEP and PEA facilitated the discriminative memory and LTP. However the direct inhibition by MPEP and a PEA-induced glutamate normalization [16] would determine an (excessive) inhibitory synergism on excitatory synaptic transmission with detrimental effect on discriminative memory [50,58,59]. Moreover, the effect of PEA in rescuing memory is associated with an increase in 2-arachidonoylglycerol (2-AG) level and a 2-AG-dependent synaptic potentiation observed at LEC-DG pathway [16,60]. Importantly, this form of LTP initiates through mGluR5 stimulation, thus the blockade of mGluR5 plays a counterproductive effect on discriminative memory. It remains to be understood why in mice chronically treated with PEA the blockade of mGluR5 did not affect LTP, whereas it caused an impairment in discriminative memory. LTP is a molecular mechanism within a single or few neural circuitries, whereas cognition is a functional process involving several circuitries, neurotransmitter and the entire brain areas, all of which are highly integrated and thus cannot be strictly correlated. Nevertheless, the discovery of the molecular mechanisms underlying the cancellation of the effect of the double treatment with PEA and MPEP, which both per se facilitated the memory in SNI mice, certainly deserves more investigations.

### 3.4. The Role of mGluR8 on the Effect of PEA on Discriminative Memory and LTP at the LEC-DG Pathway in Sham and SNI Mice

Unlike the mGluR5, the blockade of mGluR8 failed to affect the discriminative memory in either control or SNI mice. The investigation on the role of mGluR8 on cognitive behavior has so far been limited to studies using mGluR8 knockout mice, whose phenotype showed a mild [61] or no deficit [62,63] on memory and learning. mGluR8, which is highly expressed in the LEC-DG pathway, is located presynaptically outside the synaptic cleft [64] being activated under excessive amount of glutamate and playing a negative feedback action on further production, packaging, transport, and/or release of glutamate [65]. The blockade or deletion of mGluR8 may thus disrupt this regulation rendering the synapse more vulnerable to harmful glutamate excess. However, mGluR8 expression has mainly been found on GABAergic terminals [47,48,64,66], thus its blockade would lead to an increase of GABA level counteracting the glutamate elevation and the LTP/cognition alterations. Moreover, in SNI mice chronically treated with PEA, the blockade of mGluR8 did not change the effects of PEA on discriminative memory. The importance of the mGluR8 in regulating neurotransmitter levels would appear superfluous in SNI mice chronically treated with PEA in which the glutamate levels have been normalized [16] and would justify the lack of the effect of MDCPG on cognitive behavior. Alternatively, a downregulation of mGluR8 expression by PEA acting on peroxisome proliferator-activated receptor alpha (PPAR-α), a nuclear receptor protein that acts as transcription factor regulating protein expression similarly to glucocorticoids, or the absence of a tonic role for mGluR8 in the DG, cannot be excluded and deserves further investigations. Intriguingly, a single administration of MDCPG did not changed the fEPSPs in sham mice, whereas it increased both the amplitude and the slope of basal fEPSPs, thus saturating and preventing the further potentiation after TBS in SNI mice. This is in line with the observation that MDCPG, even if administered at a lower concentration than that used in the current study, failed to modify fEPSP amplitude in the LEC-DG pathway in hippocampal slices from normal rats [67]. However, MDCPG prevented the LTP induction in SNI mice chronically treated with PEA. The effect of MDCPG in increasing the amplitude and slope of the basal fEPSPs, which was still present in SNI mice chronically treated with PEA, would hinder a further potentiation and LTP plausibly through a chemically-induced saturation of amplitude and slope of basal fEPSPs. As already seen for MPEP, the combined treatment with PEA and MDCPG also shows discrepancy between the effects on memory and fEPSPS. On one hand, the LTP represents the molecular basis of memory, but on the other hand it is reductive to compare a process so integrated and complex with the activity of a single/few synapses. There are several reports where changes in memory performance were not accompanied by changes in LTP and vice versa, or where memory changes and LTP were even negatively correlated [68,69,70,71]. Several reasons may justify this apparent discrepancy: (1) a different sensitivity of LTP and cognitive behavior to the molecular changes following receptor blockade; (2) the use of inappropriate or insensitive protocols; (3) the fact that the effect on cognitive behavior involves a sum of neurotransmitters, neural patterns and brain regions, while the effect on LTP involves few synaptical connections; (4) learning, but not LTP can be modulated by non-cognitive factors such as a higher motivational drive. However, the increase in the synaptic strength by mGluR8 blockade is in agreement with the presumed function of this mGluR subtype of exerting presynaptic inhibition and points further to a role of mGluR8 in keeping the excitation/inhibition balance, which is one of the most critical variables in the processing of sensory information, and of pivotal importance in pain-related neuropsychiatric disorders.

## 4. Materials and Methods

### 4.1. Animals

Male C57BL/6J mice (20–25 g, Envigo, Italy) were housed under controlled conditions (12 h light/dark cycle, 20–22 °C ambient temperature, 55–60% humidity and *ad libitum* chow and tap water) for at least one week before the commencement of experiments. All experimental procedures were approved by the Animal Ethics Committee of University of Campania “*L. Vanvitelli*”, 1050-2016-PR, 31 October 2016 Organismo Preposto al Benessere Animale, OPBA. Animal care was in compliance with Italian (D.L. 116/92) and European Commission (O.J. of E.C. L358/1 18/12/86) regulations on the protection of laboratory animals. All efforts were made to reduce both animal numbers and suffering.

### 4.2. Spared Nerve Injury

Neuropathic pain was induced according to the method of Decosterd and Woolf [72]. Mice were anaesthetized with sodium pentobarbital (50 mg/kg, i.p.), and the tibial and common peroneal components of the sciatic nerve were tightly ligated (5.0 silk thread) and transected leaving the sural component intact. Sham mice were anesthetized, the sciatic nerve was exposed at the same level, but not ligated.

### 4.3. Treatment

The chronic treatment with vehicle or um-PEA (10 mg/kg, i.p. once a day) started 15 days after the sham or SNI surgery (day 0) and lasted for 15 days. Sham and SNI mice were tested 30 days after surgery. In the novel object recognition test a single administration of MPEP (1 mg/kg i.p.) or MDCPG (50 mg/kg i.p.) has been performed systemically 30 days after the sham or SNI surgery, 15 days after the treatment with vehicle or um-PEA and 60 min before the acquisition trial of the novel object recognition as described below. For the electrophysiological experiments, a single microinjection of MPEP (5 nmol) or MDCPG (100 nmol) was locally microinjected into the LEC at the same time point (30 days after the sham or SNI surgery and 15 days after the treatment with vehicle or um-PEA) and 15 min after the registration of the fEPSP baseline. A schematic illustration showing the timeline of surgery, treatment and experiments in sham and SNI mice is shown in Figure 1.

### 4.4. Novel Object Recognition

Mice were subjected to 1 h habituation period for exploring the apparatus, consisting of a gray polyvinyl chloride open box (40 × 30 × 30 cm; width × length × height) illuminated by a dim light. The day after, each mouse was allowed to explore two identical objects for 5 min (acquisition) and 2 h after the acquisition, one of the two objects was replaced with a new one object. The exploring time was considered as the time the mouse spent with its nose directed, and within 1 cm from the object. The behavior was recorded and analyzed by a video camera (Any-maze, Stoelting Co., Wood Dale, IL, USA). Data were expressed as recognition index (R.I.): the percentage of the time the mouse spent exploring the novel object/ the time the mouse spent exploring the novel object + the time the mouse spent exploring the familiar object.

### 4.5. Surgical Preparation for In Vivo Field Potential Recordings at LEC-DG Pathway

Mice were anesthetized with urethane (1.5 g/kg, i.p.) and fixed in a stereotaxic device (David Kopf Instruments, Tujunga, CA, USA). Body temperature was maintained at 37 °C with a heating pad (Harvard Apparatus Limited, Edenbridge, Kent). Two holes were drilled in the skull over the recording (DG, AP: −2.1 mm from bregma, L: 1.5 mm from midline and V: 1.2 mm below dura) and stimulation (LEC, AP: −4.0 mm from bregma; L:, 4.5 mm from midline and V: 2.9 mm below the dura) sites according to the atlas of Franklin and Paxinos [73] and contralateral with respect to the nerve insult. The stimulating electrode was custom-designed for simultaneously stimulating and administering vehicle or drug solutions into the LEC. The stimulating and recording (tungsten 1–5 MOhm) electrodes were lowered until a fEPSP induced by test pulses (0.2 ms in duration delivered at the frequency of 0.033 Hz) was felt. Baseline was recorded for 30 min and a TBS, consisting of six trains, six bursts, six pulses at 400 Hz, interburst interval: 200 ms, intertrain interval: 20 s, was applied in the LEC in order to stimulate the perforant path (PP) fibers for inducing LTP. LTP was considered as an increase in the amplitude and slope of the fEPSPs that exceeded the baseline by 20%, and lasted for at least 30 min from the TBS. After TBS, the recording of the fEPSPs continued for 90–120 min. Signals were acquired and analyzed by WinLTP software. Electrodes placement was confirmed by histological controls. Vehicle or drugs were administered in the LEC by connecting the stimulating electrode to a polyethylene tube associated with to a SGE 1 μL syringe. Volumes of 600 nL of vehicle or drug solutions were injected over a period of 60 s.

### 4.6. Drugs

Um-PEA (0.8–6.0 μm) was kindly provided by EPITECH Group SpA, Saccolongo (PD). MPEP hydrochloride was purchased from Tocris Bioscience (Bristol, UK). MDCPG was purchased from Hello Bio (Bristol, UK). Drugs were dissolved in 0.05% dimethylsulfoxide in artificial cerebrospinal fluid (ACSF) on the day of the experiment. The dose of drugs was chosen according to our and otherin vivoandex vivostudies [17,18,27,33,47,67,74,75,76].

### 4.7. Statistical Analysis

Data were represented as mean ± SEM. Behavioral and electrophysiological data were analyzed by using two-way ANOVA for repeated measures, followed by the Bonferroni post hoc test for comparisons between groups and one-way ANOVA, followed by Dunnett’s multiple comparison post hoc test for multiple comparisons within groups. Moreover, the unpaired t-test was used for single comparison within the group. *p* values < 0.05 were considered statistically significant. Statistical analysis was carried out using Prism/Graphpad (GraphPad Software, Inc.) software which has calculated the F (degrees of freedom) and *t* (*t*-student) related to the number of animals per group (t5).

## 5. Conclusions

These preliminary data suggest that a chronic treatment with PEA or a single administration with mGluR5 blockers (or a combination of both) affect discriminative memory and LTP at the LEC-DG pathway in neuropathic pain conditions, in which a decline of LTP and discriminative memory was observed. The mGluR8 blockade, which is ineffective per se, prevents the beneficial effect of PEA on LTP in SNI conditions.The discovery of the molecular mechanisms which make these receptors necessary for the effects of PEA may open the way to the discovery of novel multitarget therapies against cognitive decline associated with chronic pain conditions.
